# On the Endoscope

**Published:** 1868-12

**Authors:** 


On the Endoscope.—The most important revelation of the endoscope is the complete establishment of the fact that the mucous membrane of the urethra, like that of the eye, is subject to a *granular* inflammation, and that this disease constitutes the true lesion in many chronic gonorrhœas. Some years ago experiments were made upon women hired for the purpose, which showed that granular ophthalmia might be inoculated into the urethra, and there produce a disease which could not be distinguished from chronic gonorrhœa.

In fact, true chronic gonorrhœa is a *granular* inflammation, identical in nature with that which produces granular inflammation of the eyelids, of the cervix uteri, and of the larynx; and one may be produced from the other by inoculation.

When *granular*, or true chronic gonorrhœa, exists, it has no tendency to spontaneous recovery, but will last an indefinite number of years, unless properly treated, and will communicate contagion as long as it continues. This disease often persists long after the patient believes himself or herself free from the clap, and hence the frequency with which men contract gonorrhœa from females whom they believe, and who believe themselves, to be free from the disease. The chief use of the endoscope is in the diagnosis and treatment of this disease. Granular inflammation of the urethra, like that of the eyelids and cervix uteri, is extremely slow and obstinate, sometimes requiring several months, or even a year or more, to effect a cure. As long as the endoscope shows any granulations, the disease and its contagiousness continue. During all this time the discharge may be scarcely worthy of notice, and not at all purulent, but a slight irritation serves to arouse the granulations into activity, so that the patient, without being subjected to any new contagion, may have all the symptoms of a new attack of acute gonorrhœa. Chronic or granular urethritis may also be communicated in the quiescent, or more latent form, without the patient ever passing through the symptoms of ordinary manifest clap. Hence many persons will give gonorrhœa to others, who are utterly unaware that they have had the disease themselves. In the French and Prussian official examinations, the quiescent form of the disease has until lately escaped notice; for granulations were not looked for, nor appreciated, but only the more noticeable phenomena of ordinary gonorrhœa and syphilis. Hence the license system for a long time proved very inefficient in restraining the spread of gonorrhœa.

The tendency of the granular disease is also gradually to infiltrate the mucous membrane with permanently organized plastic lymph, and thus lead to stricture. Hence the vulgar opinion that stricture is generally due either to the acuteness of the first inflammation, or to the injudicious use of nitrate of silver and other injections, must be considered erroneous. Granular urethritis tends towards stricture in all cases, though the narrowing is not always so great as to trouble the patient's urination.—*N. Y. Medical Gazette.*

